# Tuberculosis

**Published:** 1899-03

**Authors:** 


### TUBERCULOSIS.

A special commission of the New York Senate has made a report on the spread of tuberculosis. Dr. Arthur B. Guerard, who made an investigation of the condition of the Fourth and Sixth Wards of New York City, made an interesting and somewhat sensational report. In the Fourth Ward, with a population slightly in excess of 18,000 and 663 dwellings, consumption was found in 248 of these houses. Of 62,000 dwellings in the entire city the proportionally large number of 19,000 were inhabited by persons afflicted with tuberculosis during the last five years.—*Medical Arena* for March, 1899.

